# Intrinsic default—executive coupling of the creative aging brain

**DOI:** 10.1093/scan/nsz013

**Published:** 2019-02-20

**Authors:** Areeba Adnan, Roger Beaty, Jaeger Lam, R Nathan Spreng, Gary R Turner

**Affiliations:** 1 Department of Psychology, York University, Toronto, Ontario Canada; 2 Department of Psychology, Harvard University, Cambridge, Massachusetts, USA; 3 Laboratory of Brain and Cognition, Montreal Neurological Institute and Department of Neurology and Neurosurgery, McGill University, Montreal, Quebec, Canada; 4 Departments of Psychology and Psychiatry, McGill University, Montreal, Quebec, Canada

**Keywords:** creativity, default mode network, aging, executive function

## Abstract

Creativity refers to the ability to generate novel associations and has been linked to better problem-solving and real-world functional abilities. In younger adults, creative cognition has been associated with functional connectivity among brain networks implicated in executive control [fronto-parietal network (FPN) and salience network (SN)] and associative or elaborative processing default network (DN). Here, we investigate whether creativity is associated with the intrinsic network architecture of the brain and how these associations may differ for younger and older adults. Young (mean age: 24.76, *n* = 22) and older (mean age: 70.03, *n* = 44) adults underwent multi-echo functional magnetic resonance image scanning at rest and completed a divergent-thinking task to assess creative cognition outside the scanner. Divergent thinking in older adults, compared to young adults, was associated with functional connectivity between the default and both executive control networks (FPN and SN) as well as more widespread default–executive coupling.

Finally, the ventromedial prefrontal cortex appears to be a critical node involved in within- and between-network connectivity associated with creative cognition in older adulthood. Patterns of intrinsic network coupling revealed here suggest a putative neural mechanism underlying a greater role for mnemonic processes in creative cognition in older adulthood.

## Introduction

Creativity is commonly defined as the ability to produce something novel and useful (Stein, 1953). It is critical for social and economic development but also for almost all areas of daily living (Duhamel, 2016). Creativity has been measured using divergent-thinking measures, since it was operationalized as a construct by Guilford (1950). Creativity is a predictor of academic and career success (Torrance, 1988; Plucker, 1999) and is positively associated with problem-solving abilities (Furnham and Bachtiar, 2008). Positive associations between creativity and functional independence have been observed in older adulthood (Duhamel, 2016). Early work examining divergent thinking, a common measure of creativity, in older adulthood suggested a progressive decline in creative cognition commencing in middle age (Alpaugh and Birren, 1977; Jaquish and Ripple, 1984; Reese *et al.*, 2001). However, this pattern of decline may also be explained by age-related declines in fluid cognitive abilities such as working memory (Roskos-Ewoldson *et al.*, 2008) or processing speed (Foos and Boone, 2008) rather than reduced creative ability *per se*.

More recent findings have failed to identify an age-related decline in creativity (Foos and Boone, 2008; Roskos-Ewoldson *et al.*, 2008; Addis *et al.*, 2016; Madore *et al.*, 2016; Palmiero *et al.*, 2014). These studies observe similar levels of creativity between younger and older adults but speculate that the cognitive substrates of creativity may change with age. One hypothesis suggests that creative cognition becomes increasingly reliant on semantics or crystalized knowledge that is relatively preserved into older age (Palmiero *et al.*, 2014). Consistent with this idea, older adult performance on a divergent-thinking task benefited from a pre-task episodic simulation exercise involving recollection of a personal past event (Madore *et al.*, 2016). The authors interpreted this as reliance on mnemonic processes to support creativity in older adults.

In recent years, the field of creativity neuroscience has focused on the neural substrates of creative cognition. The vast majority of reports has employed functional magnetic resonance image (fMRI) methods to record brain activity in younger adult subjects during performance on measures of divergent thinking (Fink *et al.*, 2006, 2009, 2010; Abraham *et al.*, 2012; Cousijn *et al.*, 2014; Kleibeuker *et al.*, 2013, 2017). The pattern of task-based brain activity associated with creative cognition in these studies closely overlaps two canonical functional brain networks, the default network (DN) and the fronto-parietal network (FPN).

The DN, including in part the inferior parietal lobe, pos-terior cingulate cortex (PCC), and middle temporal gyrus, has been consistently implicated in divergent thinking (Gonen-Yaacovi *et al.*, 2013), particularly in the early generative phases of task performance (Beaty *et al.*, 2015, 2016). Regions of the FPN, including the right dorsolateral prefrontal cortex, are hypothesized to be important in exerting cognitive control during the latter idea-evaluation phase (Benedek *et al.*, 2014; Beaty *et al.*, 2015; Chen *et al.*, 2015, 2017; Wu *et al.*, 2015). While executive control and DNs can demonstrate an antagonistic relationship during cognitive control tasks, recent work suggests that they positively couple during control tasks when access to prior knowledge is congruent with task goals (Spreng and Schacter, 2012; Spreng *et al.*, 2014). Recent investigations of functional connectivity in young adults performing various creative tasks and domains reveal a pattern of default–executive coupling that was positively associated with task performance (Jung *et al.*, 2013; Green, 2016; Mayseless *et al.*, 2015; Zabelina and Andrews-Hanna, 2016; Beaty *et al.*, 2016; Christoff *et al.*, 2016). While speculative, this pattern of functional coupling is consistent with behavioral evidence suggesting that access to prior knowledge, mediated by DN brain regions, can support creative cognition (Madore *et al.*, 2015, 2016).

The salience network (SN) has also been shown to couple with the default and executive control networks during creative cognitive tasks (Beaty *et al.*, 2015). The SN has been implicated in the detection of behaviorally relevant stimuli and redirecting attentional resources to salient stimuli in one’s external or internal milieu (Uddin, 2015). Two nodes of the SN, the dorsal anterior cingulate cortex and anterior insula, are important for creative cognition. Interestingly, both FPN and SN nodes are interconnected and have been postulated to form a broader executive control network (Dosenbach *et al.*, 2007). During divergent-thinking tasks, the DN shows dynamic coupling with the executive control network (SN and FPN) at different phases of creative thought (Beaty *et al.*, 2015). In the early, generative phase coupling is increased between the DN and SN. In the latter, evaluative phase of the task DN coupling shifts from SN to FPN regions (Beaty *et al.*, 2015). Recent work from our group observed a similar pattern of default–executive control coupling that was greater for older *vs* younger adults, despite equivalent performance on the divergent-thinking task (Adnan *et al.*, 2019). Here, we extend beyond task activation paradigms to examine the relationship between creativity and the intrinsic functional architecture of the brain in older and younger adults.

Patterns of functional connectivity observed in the brain during the resting state may be shaped by the repeated entrainment of functional connections associated with cognitive processing (Aziz-Zadeh *et al.*, 2012; Dietrich and Kanso, 2010; Stevens and Spreng, 2014; Wei *et al.*, 2014; Zhao *et al.*, 2014). Resting state functional connectivity (RSFC) measures have been associated with numerous cognitive abilities and are increasingly investigated as putative neural markers for cognitive functioning in health and disease (Fox and Raichle, 2007). Consistent with this idea, creative thought has been associated with greater static and dynamic connectivity among hubs of the default and executive networks at rest (Beaty *et al.*, 2014; Zhu *et al.*, 2017; Sun *et al.*, 2018; Beaty *et al.*, 2018a). This connectivity pattern has also been positively associated with creative cognition outside the scanner (Beaty *et al.*, 2018a). A similar pattern of network coupling has also been associated with the personality trait of ‘openness’, reflecting individual differences in one’s tendency to engage in imaginative and creative processes (Beaty *et al.*, 2018b).

Our recent task-based findings suggest that greater coupling between default and executive control systems may support creative thought in older adulthood (Adnan *et al.*, 2019). As discussed above, the pattern of functional connectivity we observed is consistent with a recent report suggesting that older adults show greater reliance on mnemonic processes, associated with DN functioning, during creative task performance (e.g. Madore *et al.*, 2016). Similarly, engagement of executive control regions has been shown to be modulated by the level of complexity in creativity tasks (Beaty *et al.*, 2015). As older adults are known to over-recruit executive control brain regions at lower levels of task demand (Reuter-Lorenz and Cappell, 2008), it follows that these patterns of greater default and executive network activity during creative cognition may be functionally coupled. This idea is consistent with the default–executive coupling hypothesis of aging (DECHA; Turner and Spreng, 2015; Spreng and Turner, 2019). The DECHA suggests that functional connectivity between these two networks is a core feature of neurocognitive aging and may support cognitive performance when access to prior knowledge is congruent with task goals (Spreng and Schacter, 2012; Turner and Spreng, 2015; Spreng *et al.*, 2018). Enhanced default–executive coupling observed during a divergent-thinking task would be consistent with the idea that access to prior knowledge may support creative thought in older adults (Madore *et al.*, 2016; Palmeiro *et al.*, 2014).

Whether this pattern of altered functional connectivity represents a task-specific alteration in brain networks implicated in creative thought or reflects a more enduring shift in the intrinsic connectivity of the brain in later life has yet to be investigated. Here, we use RSFC MRI to investigate whether patterns of RSFC within and among brain networks implicated in creative cognition predict creativity measured outside of the scanner and whether observed brain and behavioral associations differ by age. Consistent with the DECHA model, we predict that intrinsic coupling between regions of the default and executive control networks will be associated with better performance on a divergent-thinking task and that this association would be more robust in older *vs* younger adults.

## Methods

### Participants

Young and older adults were recruited from the community and completed a comprehensive cognitive test battery and MRI scanning as part of a larger ongoing multi-site study at York University and Cornell University. Included were 32 older adults in the current study from York University while 12 older adults were included from Cornell University, giving a final sample of 44 older adults (mean age = 70.03 years, s.d. = 4.75; 21 females). 18 young adults from York University, and 4 young adults from Cornell University were included in the sample. The final sample comprised of 22 younger adults (mean age: 24.76, s.d. = 3.36; 15 females) that were included in the current study. Of note, females were overrepresented in the final samples for both age groups and slightly more so in the younger adult sample. There was no difference in creativity between men (*M* = 2.79, s.d. = 0.55) and women (*M* = 2.68, s.d. = 0.4), *F*(1, 62) = 0.37, *P* = 0.55 and between younger (*M* = 2.81, s.d. = 0.38) and older (*M* = 2.59, s.d. = 0.53) adults, *F*(1, 62) = 3.3, *P* = 0.07. Furthermore*,* previous research has failed to find evidence for sex effects in creativity (Reese *et al.*, 2001), suggesting that this difference should not impact the interpretability or generalizability of the findings. Participants received monetary compensation for their time (equivalent to $50 CAD/USD for the MRI scan and $10 CAD/USD per hour). To be eligible for the study, participants had to be (i) between the ages of 18 and 35 (Young) or over age 60 (Old), (ii) right-handed and (iii) a fluent English speaker. Exclusion criteria included any MRI contraindications and/or a history of neurological, neuropsychiatric or cardiovascular disease. All participants provided informed consent consistent with procedures approved by the Institutional Review Boards of York University and Cornell University. All participants were cognitively normal based on self-report on intake and cognitive screen [Mini Mental State Exam (MMSE) > 26].

Previous work has shown that both creative ability (McCrae, 1987; Feist, 1998; Silvia *et al.*, 2009) and DN engagement (Beaty *et al.*, 2018b) are predicted by the ‘Big-Five’ personality trait of ‘openness’. All participants completed both the divergent-thinking measures and a comprehensive personality inventory, the Big Five Aspect Scales (BFAS; Goldberg, 1992). A two-tailed *t*-test revealed that there was a significant difference in self-reported openness to experience between young (*M* = 3.52, s.d. = 0.26) and older (*M* = 3.81, s.d. = 0.24) adults, *t*(64) = −2.19, *P* = 0.032, Cohen’s *d* = 1.16. Furthermore, openness to experience was significantly correlated with creative ratings across all participants [*r*(62) = 0.26, *P* = 0.03], in older adults [*r*(42) = 0.39, *P* = 0.008] and in young adults [*r*(20) = 0.44, *P* = 0.04]. Given prior work associating creativity and openness and recent investigations showing that intrinsic networks associated with creativity also co-vary with openness to experience (Beaty *et al.*, 2018b), we used BFAS-openness to experience (BFAS-O) as a control variable in all subsequent analyses.

### Offline measures of creative ability

The divergent-thinking task was completed by all participants outside of the scanner and consisted of three paper–pencil alternate uses tasks (Kaufman *et al.*, 2008). The alternate uses tasks required participants to generate creative uses for three common objects: a box, a rope and a knife. Participants had three minutes to verbally articulate as many responses as possible, which were recorded by the test administrator. After each task, participants were presented with their list of responses and asked to rank them for creative quality. Ranking permits the use of a top-scoring method wherein the originality score is based on the creativity evaluation of a predefined number of top ideas (Silvia *et al.*, 2008). The top-scoring method addresses confounds of fluency and ‘represents people’s best efforts, in their own judgment, and it thus represents people’s best level of performance when they are instructed to do their best (p. 71).’ In addition, the top-scoring method has a psychometric benefit of standardizing the number of responses across participants.

Participant-identified top ideas were then scored by three trained raters who were blind to participants’ age group (Christensen *et al.*, 1957; Silvia *et al.*, 2008; Benedek *et al.*, 2013). The three raters were trained to score responses for creative quality, using a 1 (not at all creative) to 4 (very creative) scale. We applied the Top 3 scoring procedure (Silvia *et al.*, 2008; Benedek *et al.*, 2014) involving selection of the three most creative responses indicated by participant rankings and averaged across the three raters’ scores. Overall creativity ratings were obtained by averaging ratings for each of the three common objects.

There was a moderate level of convergence between ratings provided by raters for the three tasks. The inter-rater reliability between the three raters was interclass coefficient (*ICC*) = 0.62, 0.59, 0.61 for the tasks ‘box’, ‘rope’ and ‘knife’, respectively. This level of moderate inter-rater reliability is consistent with previous reports and aligns with the overall literature employing this scoring method (Benedek *et al.*, 2013). We also computed inter-rater reliability for responses generated by young and older adults. There was moderate inter-rater reliability observed between raters for older adults, (*ICC* = 0.51, 0.57, 0.53) and for young adults (*ICC* = 0.56, 0.61, 0.59) for the tasks `box’, `rope’ and `knife’. There was no significant difference in creative ability as measured by average ratings between young (*M* = 2.79, s.d. = 0.3) and older (*M* = 2.59, s.d. = 0.14) adults, *t*(42) = 1.39, *P* = 0.17, Cohen’s *d* = 0.85. Thus, older adults provided similarly creative ideas as their younger counterparts.

### RSFC analyses

#### Multi-echo fMRI data acquisition and pre-processing

Imaging data for participants recruited at Cornell University were acquired using 3T GE Discovery MR750 scanner (General Electric, Milwaukee, USA) with a 32-channel receive-only phased-array head coil at the Cornell Magnetic Resonance Imaging Facility in Ithaca. Imaging data for participants recruited at York University were acquired using a Siemens 3T Magnetom Tim Trio MRI scanner. All scanning protocols were carefully matched across sites.

Anatomical scans from the Cornell MRI Facility were acquired with a T1-weighted volumetric MRI magnetization-prepared rapid gradient echo [repetition time (TR) = 2530 ms; echo time (TE) = 3.44 ms; flip angle (FA) = 7°; 1.0 mm isotropic voxels, 176 slices]. Anatomical scans were acquired during one 5 min 25 s run with 2× acceleration with sensitivity encoding. Anatomical scans from the York University MRI Facility were acquired with a T1-weighted volumetric MRI magnetization-prepared rapid gradient echo (TR = 900 ms; TE = 2.52 ms; TI = 900 ms; FA = 9°; 1.0 mm isotropic voxels, 192 slices). Anatomical scans were acquired during one 4 min 26 s run with 2× acceleration with generalized autocalibrating partially parallel acquisition (GRAPPA) encoding with an integrated parallel imaging techniques (iPAT) acceleration factor of 2. Structural data was corrected for non-uniform intensities, affine registered to Montreal-Neurological Institute (MNI) atlas and skull-stripped using FMRIB Software Library (FSL).

**Table 1:** ROI-to-ROI connectivity positively correlated with divergent-thinking ability in young adults (corresponding to Figure 1)

		**Network**	**Hem**	**Node**	**MNI coordinates**			***T***	***P***
					***X***	***Y***	***Z***		
**Young adults**									
***Between-network connectivity***									
***SN–FPN***									
**IFG**		FPN	L	109	−43	19.4	33.5		
	mACC	SN	L	28	−9	25.3	27.7	3.94	0.01
	Anterior Insula	SN	L	84	−28.8	23.7	8.4	3.37	0.03
***DN–FPN***									
**vmPFC**		DN	R	279	7.2	48.4	−10.1		
	ITG	FPN	L	9	−55.9	−47.7	−9.3	4.09	0.006
	MFG	FPN	L	108	−43	19.4	33.5	3.94	0.006
**vMPFC**		DN	L	117	−6.8	38.2	−9.4		
	DLPFC	FPN	R	328	38.9	9.6	42.7	3.41	0.04
**IFG**		FPN	R	276	38.6	18.8	25.5		
	vmPFC	DN	L	152	−6	44.9	6.3	3.91	0.013
**MFG**		FPN	L	108	−43	19.4	33.5		
	PCC	DN	L	1	−11.2	−52.4	36.5	4.27	0.004
	Medial superior PFC	DN	L	6	−47.2	−58	30.8	4.21	0.004
	vmPFC	DN	L	116	−5.9	54.8	11.3	3.98	0.01
**MFG**		FPN	L	149	28.6	50.9	10.1		
	DLPFC	DN	L	156	−29.3	16.8	50.7	3.51	0.04
***DN–SN***									
**Precentral gyrus**		SN		22	−9.4	−0.1	42.9		
	medPFC	DN	R	200	21.9	21	46.2	3.49	0.04
**ACC**		SN	L	27	−8.4	14.6	33.8		
	vmPFC	DN	L	116	−5.9	54.8	−11.3	3.57	0.01
**PCC**		DN	L	26	−1.7	−17.7	39.1		
	SFG	SN	R	181	6.7	5	55.9	3.38	0.03
	mACC	DN	R	185	8.6	4.2	40.1	3.37	0.04
	PCC	DN	R	186	3	−19.6	37.9	3.61	0.03
***Within-network connectivity***									
***SN–SN***									
**Rolandic operculum**		SN	L	101	−59.8	−4.1	8.8		
	Anterior insula	SN	L	82	−37.3	2.9	11.7	3.8	0.01
***DN–DN***									
**medPFC**		DN	R	323	5.9	54.9	29.4		
	MTG	DN	R	290	57.5	−7.4	−16.4	4.43	0.002
**vmPFC**		DN	L	152	−6	44.9	6.3		
	medPFC	DN	R	322	8.2	53.8	14	4.3	0.0036
**vmPFC**		DN	L	117	−6.8	38.2	−9.4		
	DLPFC	DN	R	165	11.9	21.9	59.9	3.3	0.004
**medPFC**		DN	R	200	21.9	21	46.2		
	medFG	DN	R	165	11.9	21.9	59.9	3.81	0.01

**Note:** ACC—anterior cingulate cortex; DLPFC—dorsolateral prefrontal cortex; DN—default network; FPN—fronto-parietal network; Hem—hemisphere; IFG—inferior frontal gyrus, ITG—inferior temporal gyrus; L—Left; mACC—middle anterior cingulate cortex; medFG—medial frontal gyrus; medPFC—medial prefrontal cortex; PCC—posterior cingulate cortex; R—right; SFG—superior frontal gyrus; SN—salience network; vmPFC—ventromedial prefrontal cortex.

**Fig. 1:**
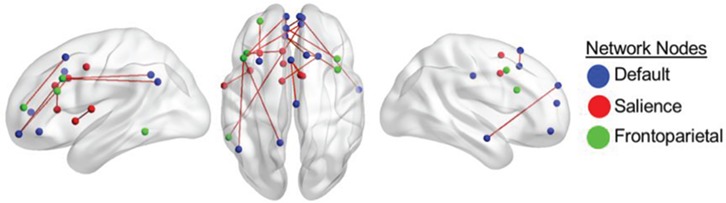
RSFC correlating with divergent-thinking ability in young adults after controlling for scanner site and personality (openness to experience). Color-coded nodes include regions from the DN, FPN and SN. The color of the edges denotes the direction of correlation between functional connectivity and divergent-thinking ability. Only positive correlations between ROI-to-ROI functional connectivity and divergent-thinking ability survived a seed-level false discovery rate (FDR) correction at an alpha level of 0.05. Results correspond to findings in Table 1.

**Fig. 2:**
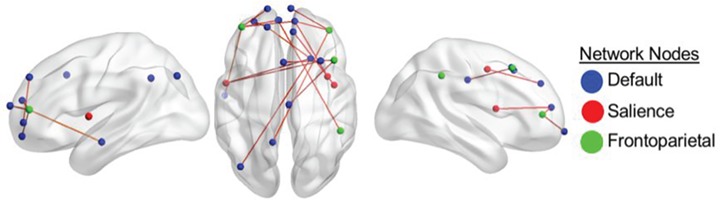
RSFC correlated with divergent-thinking ability in older adults after controlling for scanner site and personality (openness to experience). Color-coded nodes include regions from the DN, FPN and SN. The color of the edges denotes the direction of correlation between functional connectivity and divergent-thinking ability. Only positive correlations between ROI-to-ROI functional connectivity and divergent-thinking ability survived a seed-level FDR correction at an alpha level of 0.05. Results correspond to findings in Table 2.

Multi-echo fMRI was developed as a data acquisition sequence to facilitate removal of noise components from resting fMRI data sets (Kundu *et al.*, 2012, 2013; Power *et al.*, 2018). This acquisition method can lead to a 4-fold improvement in the temporal signal-to-noise ratio in resting-state fMRI (Kundu *et al.*, 2015) and has been found to effectively remove distance-dependent motion confounds in RSFC analyses (Power *et al.*, 2018). The method relies on the acquisition of multiple echoes, allowing direct measurement of T2^*^ relaxation rates. Blood oxygen level–dependent (BOLD) signal can then be distinguished from non-BOLD noise on the basis of TE dependence. The multiple TEs are recombined and analyzed using independent components analysis (ICA) to remove noise components (such as those originating from white matter, cerebrospinal fluid (CSF), movement). This method has shown to be successful in denoising BOLD signal of motion and physiological artifacts (Kundu *et al.*, 2012, 2013). Participants completed one 10 min 6 s resting-state multi-echo (ME) BOLD functional scans with eyes open, blinking and breathing normally in the dimly lit scanner bay. At Cornell University, resting-state functional scans were acquired using a ME echo planar imaging (ME-EPI) sequence with online reconstruction [TR = 3000 ms; TEs = 13.7, 30, 47 ms; FA = 83°; matrix size =72 × 72; field of view (FOV) = 210 mm; 46 axial slices; 3.0 mm isotropic voxels]. Resting-state functional scans were acquired with 2.5× acceleration with sensitivity encoding. At York University, resting-state functional scans were acquired using a ME-EPI sequence with online reconstruction (TR = 3000 ms; TEs = 14, 30, 46 ms; FA = 83°; matrix size = 64 × 64; FOV = 216 mm; 43 axial slices; 3.4 × 3.4 × 3 mm voxels). Resting-state functional scans were acquired with 3× acceleration with GRAPPA encoding. Data were pre-processed with ME-ICA version 2.5 (https://afni.nimh.nih. gov/pub/dist/src/pkundu/meica.py) and aligned to MNI space**.** ME-ICA processing was then run with the following options: −e 13, 30, 46, −b 15 s; –no_skullstrip; –space = Qwarp_meanE + tlrc. Qwarp_meanE + tlrc represented an averaged MNI-space template of our younger and older adults. As we were interested in functional brain networks, smoothing was not applied as this has been shown to artificially affect the similarity of networks across subjects (Alakörkkö *et al.*, 2017). Data were not further filtered as ME-ICA has shown to be successful in denoising BOLD signal of artifacts (Kundu *et al.*, 2012, 2013). Components identified as both noise and signal were visually inspected for further quality control. Accepted components identified as signal were compiled in a single 4D file to be used for further connectivity analyses.

#### Resting-state network functional connectivity matrices

Regions of interest (ROIs) for the FPN, DN and SN were defined using the network parcellation scheme by Gordon *et al.* (2014). In total, we used 105 ROIs (40 SN; 41 DN; 24 FPN).

The CONN toolbox (Whitfield-Gabrieli and Neito-Castanon, 2012) was used to examine ROI-to-ROI functional connectivity. The mean time series of voxels within each of the 105 ROIs was averaged across the resting-state run and correlated with the average time series of all other ROIs. Resulting Pearson correlation coefficients were then fisher-to-z transformed and are referred to as functional connectivity in analyses (detailed below in *Analysis Approach*). Given that this was a multi-site study, we included scanner location as a nuisance regressor in all analyses.

#### Analysis approach

##### Within-group RSFC associated with creative ability

First, we used a within-group approach to examine creativity-associated patterns of RSFC among our networks of interest in young and older adults, while controlling for openness to experience and scanner site. Here, we examined within group patterns of connectivity (young and older adults) independently, and offline measures of creativity were used as a second-level regressor of interest in both analyses. Functional connectivity between all possible ROI pairs was tested using individual level *t*-tests, between each seed and target ROI pair. Results were corrected for multiple comparisons using a false discovery rate threshold of 0.05 at the ROI level. For both groups, positive findings reflect patterns of ROI-to-ROI connectivity that positively correlate with creative ability, while negative findings indicate negative correlations with creative ability.

##### Between-group RSFC associated with creative ability

Second, to examine age-related differences in creativity, we adopted a between group analysis. Here, we contrasted group level maps of ROI-to-ROI functional connectivity correlated with offline measures of creativity. This contrast was specified as Older Adults > Young Adults*.* Results were corrected for multiple comparisons using a false discovery rate threshold of 0.05 at the ROI level. For this analysis, positive findings reflect patterns of functional connectivity that correlate with creative ability in older adults; negative findings indicate patterns of functional connectivity that correlate with creative ability in young adults.

**Table 2:** ROI-to-ROI connectivity positively correlated with divergent-thinking ability in older adults (corresponding to Figure 2)

		***Network***	***Hem***	***Node***	***MNI coordinates***			***T***	***P***
					***X***	***Y***	***Z***		
**Older adults**									
***Between-network connectivity***									
***DN–SN***									
**medPFC**		DN	R	322	8.2	53.8			
	Superior insula	SN	R	238	36.7	5.2	12.7	3.78	0.01
**medPFC**		DN	R	325	6.8	44.5	34.8		
	FEF	SN	R	198	42.5	−2.3	47.2	3.74	0.02
**Precentral gyrus**		SN	L	111	−51.8	−0.6	5		
	medPFC	DN	R	200	21.9	21	46.2	4.25	0.0035
	MFG	DN	R	326	30.6	18.9	48.7	4.1	0.0035
***FPN–DN***									
**DLPFC**		FPN	L	7	−38.1	48.8	10.5		
	medPFC	DN	L	150	−6.5	54.7	18.1	4.17	0.005
**SFG**		FPN	R	327	42.4	19.5	48.2		
	PCC	DN	R	186	3	−19.6	37.9	4.05	0.008
	PCC	DN	L	26	−1.7	−17.7	39.1	3.91	0.01
**DLPFC**		FPN	L	7	−38.1	48.8	10.5		
	medPFC	DN	R	322	8.2	53.8	14	3.8	0.01
**MFG**		FPN	R	168	38.1	45.9	7.7		
	medPFC	DN	R	322	8.2	53.8	14	3.62	0.01
**DLPFC**		FPN	L	7	−38.1	48.8	10.5		
	Frontal pole	DN	L	151	−15.7	64.7	13.7	3.46	0.02
	vmPFC	DN	L	116	−5.9	54.8	−11.3	3.44	0.02
	ITG	DN	L	127	−53.1	−11.4	−16	2.98	0.04
**MFG**		FPN	R	168	38.1	45.9	7.7		
	vmPFC	DN	R	278	4.8	65.1	−7.1	4.08	0.007
	PCC	DN	R	1	−11.2	−52.4	36.5	3.36	0.03
**medPFC**		DN	L	150	−6.5	54.7	18.1		
	DLPFC	FPN	L	7	−38.1	48.8	10.5	3.66	0.03
	IPL	FPN	R	167	47.9	−42.5	41.5	3.4	0.03
***Within-network connectivity***									
***DN–DN***									
**AG**		DN	L	94	−39.3	−73.9	38.3		
	medPFC	DN	R	200	21.9	21	46.2	3.52	0.04
		DN	L	145	−15.9	48.6	37.2		
	medPFC	DN	R	200	21.9	21	46.2	3.32	0.04
	medPFC	DN	L	114	−27.5	53.6	0	3.28	0.04

**Note:** ACC—anterior cingulate cortex; AG—angular gyrus; DLPFC—dorsolateral prefrontal cortex; DN—default network; FEF—frontal eye fields; FPN—fronto-parietal network; Hem—hemisphere; IFG—inferior frontal gyrus, IPL—inferior parietal lobule; ITG—inferior temporal gyrus; L—left; mACC—middle anterior cingulate cortex; medFG—medial frontal gyrus; medPFC—medial prefrontal cortex; MFG—middle frontal gyrus; PCC—posterior cingulate cortex; R—right ; SFG—superior frontal gyrus; SN—salience network; vmpFC—ventromedial prefrontal cortex.

## Results

### Within-group patterns of functional connectivity associated with creative cognition

We examined the patterns of intrinsic functional connectivity that were significantly predictive of creative ability in older and young adults. We also examined the overlap in patterns of functional connectivity predictive of creativity in young and older adults. In these analyses, we controlled for the personality trait openness to experience and scanner site.

#### Young adults

Young adults showed a distributed pattern of between-network functional connectivity that positively predicted divergent-thinking performance outside of the scanner. Between-network connectivity predictive of creativity comprised of significant connections between (i) SN and FPN (left inferior frontal gyrus and left middle anterior cingulate cortex and left anterior insula), (ii) key nodes of the FPN and DNs [e.g. ventromedial prefrontal cortex (vmPFC), PCC, medial superior PFC] and (iii) DN and SN nodes.

Young adults also showed patterns of within-network connectivity, specifically between nodes of the SN (left rolandic operculum and left anterior insula) and nodes of the DN (e.g. left vmPFC and right medial PFC).

**Fig. 3:**
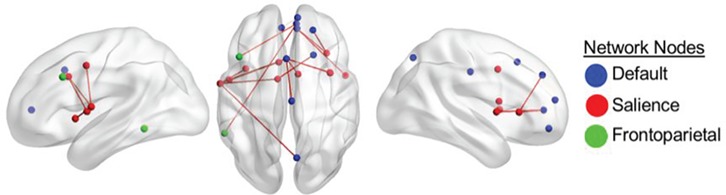
Overlap between RSFC correlated with divergent-thinking ability across young and older adults after controlling for scanner site and personality (openness to experience). Color-coded nodes include regions from the DN, FPN and SN. The color of the edges denotes the direction of correlation between functional connectivity and divergent-thinking ability. Only positive correlations between ROI-to-ROI functional connectivity and divergent-thinking ability survived a seed-level FDR correction at an alpha level of 0.05. Connections displayed are corrected a seed-level FDR correction at an alpha level of 0.05. Results correspond to findings in Table 3.

Table 1 and Figure 1 provide details for these nodes and associated connectivity results.

#### Older adults

For older adults, both within- and a more spatially distributed between-network connectivity profile were positively associated with creative task performance. Within-network connectivity was observed between (i) nodes of the SN (insula, postcentral gyrus, frontal eye fields) and DN (right medial PFC and middle frontal gyrus) and (ii) nodes of the FPN (dorsolateral PFC, superior frontal gyrus, middle frontal gyrus, inferior parietal lobule) and DN (medial PFC, vmpFC, inferior temporal gyrus, PCC). Widespread between-network connectivity was observed among core nodes of DN (e.g. between angular gyrus and medial PFC). Table 2 and Figure 2 provide details for these nodes and associated connectivity results.

#### All participants

Both younger and older adults have some overlap in patterns of intrinsic functional connectivity predictive of creativity. This was noted within-networks (within SN) and between networks (between DN and SN nodes and between DN and FPN nodes). There was also an overlap in within-network connectivity between DN nodes. Table 3 and Figure 3 provide details for these nodes and associated connectivity results.

### Age differences in patterns of functional connectivity associated with creative ability

When the brain–behavior correlation maps for both age groups were directly contrasted, controlling for BFAS-O and scanner site, a pattern of greater within-network connectivity was associated with better divergent- thinking performance for the younger cohort. Specifically, greater connectivity between DN nodes, including between (i) left vmPFC and bilateral medial PFC, left inferior temporal gyrus, left frontal pole and left superior frontal gyrus; (ii) right middle temporal gyrus and right medial PFC; and (iii) right medial PFC and left vmPFC and left medial PFC, was associated with better divergent-thinking ability for the younger participants. Young adults also had greater functional connectivity between (i) default and FPN nodes (e.g. between right vmPFC and left inferior temporal gyrus) and (ii) default and SN nodes (right PCC and right anterior cingulate cortex), positively associated with divergent-thinking ability.

**Table 3:** ROI-to-ROI connectivity positively correlated with divergent-thinking ability and overlapping between young and older adults (corresponding to Figure 3)

		***Network***	***Hem***	***Node***	**MNI coordinates**			***T***	***P***
					*X*	*Y*	*Z*		
***Between-network connectivity***									
***DN–SN***									
**Rolandic operculum**		SN	L	101	−59.8	−4.1	8.8		
	AG	DN	R	257	7.4	−69.3	49.9	4	0.008
**Insula**		SN	L	248	33.7	22.6	3.7		
	medPFC	DN	R	316	21.4	42.8	35.1	3.85	0.008
**PCC**		DN	L	26	−1.7	−17.7	39.1		
	Superior insula	SN	R	246	36.5	5.7	6	3.83	0.01
	Precentral gyrus	SN	L	111	−51.8	−0.6	5	3.58	0.03
	Superior insula	SN	R	238	36.7	5.2	12.7	3.54	0.04
**vmPFC**		DN	R	184	7.7	44.1	5.5		
	Postcentral gyrus	SN	R	274	50.1	3	3.9	3.7	0.02
	Insula	SN	L	248	33.7	22.6	3.7	3.56	0.02
**mACC**		SN	L	22	−9.4	−0.1	42.9		
	medPFC	DN	R	200	21.9	21	46.2	3.69	0.03
***DN–FPN***									
**vmPFC**		DN	R	279	7.2	48.4	−10.1		
	ITG	FPN	L	9	−55.9	−47.7	−9.3	3.57	0.03
	MFG	FPN	L	108	−43	19.4	33.5	3.46	0.03
***Between-network connectivity***									
***DN–DN***									
**vmPFC**		DN	L	152	−6	44.9	6.3		
	medPFC	DN	R	322	8.2	53.8	14	2.53	0.04
**PCC**		DN	L	26	−1.7	−17.7	39.1		
	PCC	DN	R	186	3	−19.6	37.9	3.73	0.02
	mACC	DN	R	185	8.6	4.2	40.1	3.37	0.03
***SN–SN***									
**Rolandic operculum**		SN	L	101	−59.8	−4.1	8.8		
	Anterior insula	SN	L	82	−37.3	2.9	11.7	4.33	0.0033
	mACC	SN	L	22	−9.4	−0.1	42.9	3.62	0.03
	ACC	SN	L	27	−8.4	14.6	33.8	3.33	0.04

**Note:** ACC—anterior cingulate cortex; AG—angular gyrus; DLPFC—dorsolateral prefrontal cortex; DN—default network; FEF—frontal eye fields; FPN—fronto-parietal network; Hem—hemisphere; IFG—inferior frontal gyrus, IPL—inferior parietal lobule; ITG—inferior temporal gyrus; L—left; mACC—middle anterior cingulate cortex; medFG—medial frontal gyrus; medPFC—medial prefrontal cortex; MFG—middle frontal gyrus; PCC—posterior cingulate cortex; R—right ; SFG—superior frontal gyrus; SN—salience network; vmpFC—ventromedial prefrontal cortex.

In older adults, greater between-network functional connectivity was associated with better divergent-thinking ability. Greater between-network functional connectivity, associated with better outside scanner task performance, was also observed between all three networks. This was not observed in young adults and included functional connectivity between right medial PFC, right intraparietal sulcus and left superior insula. There was also widespread functional connectivity between (i) default and FPN nodes in older adults that predicted creativity, including connections between left middle temporal gyrus and right intraparietal sulcus; (ii) FPN and SN nodes: (a) left middle frontal gyrus and right precentral gyrus and (b) left inferior parietal lobule and left precentral gyrus; and (iii) DN and SN nodes (e.g. right vmPFC and right superior insula).

There was also within-network connectivity observed among (i) SN nodes, between right superior insula and right supramarginal gyrus, left middle frontal gyrus middle, left postcentral gyrus, left middle anterior cingulate cortex and right anterior cingulate cortex; (ii) within the FPN, between right inferior frontal gyrus and the left inferior temporal gyrus and left middle frontal gyrus; and (iii) within the DN between right middle temporal gyrus and right medial PFC.

**Fig. 4:**
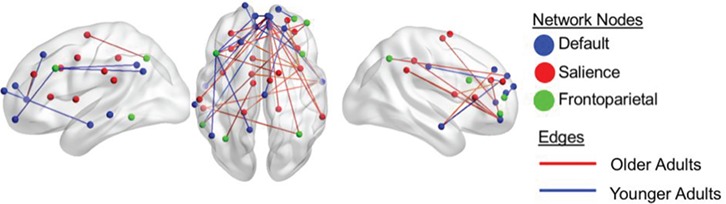
Group by behavior interaction for intrinsic connectivity correlated with divergent thinking after controlling for scanner site and personality (openness to experience). The figure shows resting-state ROI-to-ROI functional connectivity that correlates with divergent-thinking ability and is significantly different between young and older adults. Color-coded nodes include regions from the DN, FPN and SN. The color of the edges (connections between nodes) indicate the direction of the contrast. Red edges indicate greater connectivity between regions that are associated with divergent thinking in older adults, while blue edges indicate greater connectivity between regions that are associated with divergent thinking in young adults. Results correspond to findings in Table 4.

Table 4 and Figure 4 provide details for these nodes and associated connectivity results.

**Table 4:** ROI-to-ROI connectivity correlating with divergent-thinking ability contrasted between young and older adults (Older Adults > Young Adults) after controlling for the personality trait, openness to experience (BFAS-O). Here, Positive *T*-values reflect ROI-to-ROI functional connectivity predicting divergent-thinking ability in older adults compared to young adults, while negative *T-*values reflect ROI-to-ROI functional connectivity, showing a stronger association between divergent-thinking ability in young adults compared to older adults

		***Network***	***Hem***	***Node***	**MNI coordinates**			***T***	***P***
					*X*	*Y*	*Z*		
**Young adults**									
***Between-network connectivity***									
***FPN–DN***									
**MFG**		FPN		108	−43	19.4	33.5		
	PCC	DN	R	1	−11.2	−52.4	36.5	−4.23	0.004
	Medial superior PFC	DN	L	6	−11.7	26.7	57	−3.91	0.01
	vmPFC	DN	L	116	−5.9	54.8	−11.3	−3.88	0.015
**IFG**		FPN	R	276	38.6	18.8	25.5		
	vmPFC	DN	L	152	−6	44.9	6.3	−3.57	0.03
**vmPFC**		DN	R	279	7.2	48.4	−10.1		
	MFG	FPN	L	108	−43	19.4	33.5	−3.56	0.02
	ITG	FPN	L	9	−55.9	−47.7	−9.3	−3.22	0.03
**MFG**		FPN	R	320	30.9	52.2	9.9		
	medPFC	DN	R	321	16	61	19.8	−3.72	0.02
***DN–SN***									
**PCC**		DN	R	186	3	−19.6	37.9		
	ACC	SN	R	317	24.4	50.8	24.3	−3.5	0.04
***Within-network connectivity***									
***DN–DN***									
**MTG**		DN	R	290	57.5	−7.4	−16.4		
	Medial PFC	DN	R	323	5.9	54.9	29.4	−5.11	0.0002
**Medial PFC**		DN	R	323	5.9	54.9	29.4		
	medPFC	DN	L	150	−6.5	54.7	18.1	−4.14	0.02
	vmPFC	DN	L	116	−5.9	54.8	−11.3	−3.28	0.03
**vmPFC**		DN	L	152	−6	44.9	6.3		
	medPFC	DN	R	322	8.2	53.8	14	−3.85	0.02
	ITG	DN	L	127	−53.1	−11.4	−16	−3.68	0.02
	medPFC	DN	L	150	−6.5	54.7	18.1	−3.29	0.03
	Frontal pole	DN	L	151	−15.7	64.7	13.7	−3.37	0.03
	SFG	DN	L	44	−19.5	30.1	45.5	−3.23	0.03
**Older adults**									
***Between-network connectivity***									
***DN–FPN–SN***									
**Medial PFC**		DN	R	323	5.9	54.9	29.4		
	IPS	FPN	R	261	35.7	−56.7	45.2	4.01	0.009
	Superior insula	SN	L	81	−36.6	1.4	6.4	4.25	0.004
***DN–FPN***									
**MTG**		DN	L	126	−63.2	−28.7	−7.2		
	IPS	FPN	R	261	35.7	−56.7	45.2	3.35	0.03
***FPN–SN***									
**MFG**		FPN	L	108	−43	19.4	33.5		
	Precentral gyrus	SN	R	192	16.2	0.8	67.5	3.58	0.04
**IPL**		FPN	L	96	−34.1	−61	34.2		
	Precentral gyrus	SN	L	34	−8	−8.7	62.9	3.96	0.01
***DN–SN***									
**vmPFC**		DN	R	279	48.4	−10.1	−3.76		
	TPJ	SN	R	180	16.2	−33.1	43.2	4.09	0.007
	Superior insula	SN	R	249	34	24.4	10	3.73	0.007
	SMG	SN	R	219	57.5	−40.3	34.7	3.77	0.007
	MFG	SN	R	318	31.3	39.7	25.6	3.48	0.01
	Postcentral gyrus	SN	L	105	−58.8	−23.9	31	3.52	0.01
**vmPFC**		DN	L	152	−6	44.9	6.3		
	MFG	SN	L	153	−28.8	38.3	28.2	3.54	0.04
	Anterior Insula	SN	L	84	−28.8	23.7	8.4	3.67	0.03
**vmPFC**		DN	R	184	7.7	44.1	5.5		
	Precentral gyrus	SN	R	196	8	−6.2	63.7	3.52	0.04
**Superior insula**		SN	R	238	36.7	5.2	12.7		
	vmPFC	DN	R	279	48.4	−10.1	−3.76	3.34	0.01
**MTG**		DN	R	290	57.5	−7.4	−16.4		
	Postcentral gyrus	SN	L	103	−55.1	−32.3	23	3.23	0.04
	mACC	SN	L	22	−9.4	−0.1	42.9	3.15	0.04
***Within-network connectivity***									
***FPN–FPN***									
**IFG**		FPN	R	240	42.8	48.3			
	ITG	FPN	L	9	−55.9	−47.7	−9.3	4.62	0.001
	MFG	FPN	L	108	−43	19.4	33.5	3.67	0.014
***SN–SN***									
**Superior insula**		SN	R	238	36.7	5.2	12.7		
	SMG	SN	R	219	57.5	−40.3	34.7	3.33	0.01
	MFG	SN	L	153	−28.8	38.3	28.2	3.13	0.01
	Postcentral gyrus	SN	L	21	−16.6	−36.1	42.7	3.27	0.01
	mACC	SN	L	27	−8.4	14.6	33.8	3.1	0.01
	ACC	SN	R	317	24.4	50.8	24.3	2.84	0.03
***DN–DN***									
**MTG**		DN	R	290	57.5	−7.4	−16.4		
	medPFC	DN	R	316	21.4	42.8	35.1	3.23	0.04
**vmPFC**		DN	L	152	−6	44.9	6.3		
	medPFC	DN	R	323	5.9	54.9	29.4		

**Note:** ACC—anterior cingulate cortex; AG—angular gyrus; DLPFC—dorsolateral prefrontal cortex; DN—default network; FEF—frontal eye fields; FPN—fronto-parietal network; Hem—hemisphere; IFG—inferior frontal gyrus, IPL—inferior parietal lobule; IPS—intraparietal sulcus; ITG—inferior temporal gyrus; L—left; mACC—middle anterior cingulate cortex; medFG—medial frontal gyrus; MTG—middle temporal gyrus; medPFC—medial prefrontal cortex; MFG—middle frontal gyrus; PCC—posterior cingulate cortex; R—right ; SFG—superior frontal gyrus; SN—salience network; TPJ—temporo-parietal junction; vmpFC—ventromedial prefrontal cortex.

Finally, as a further check on our approach to include the BFAS-O scores as a nuisance regressor in the regression model, we performed the above analysis on a subsample of older adults (*N* = 22) matched on BFAS-O scores with young adults. The results of these analyses closely overlap the core findings described above (Supplemental Materials, Figure S1 and Table S1).

## Discussion

We investigated whether creative ability, measured as performance on a divergent-thinking task, was related to connectivity among the DN, FPN and SN and how patterns of RSFC associated with creativity differed between young and older adults. Both groups demonstrated equivalent performance on the divergent-thinking task. However, unique intrinsic functional connectivity profiles were associated with creative ability in the two age groups. Older adults had a pattern of greater functional connectivity between default and the broader executive control network, with connections observed between core nodes of the DN, FPN and SN that was associated with creativity. In contrast, while young adults showed default–executive coupling, it was limited to connectivity between default and sub-networks of executive control (e.g. between DN and SN, DN and FPN and FPN and SN) rather than connectivity among all three networks.

Several studies of younger adults have investigated the relationship between neural network properties of the brain at rest and creative ability. Creative ability has been observed to be correlated with RSFC between the PCC and medial PFC, core nodes of the DN (Takeuchi *et al.*, 2011). Performance on a common measure of creative cognition, divergent thinking, has been also associated with greater connectivity within the DN, as well as connectivity between default and executive networks (Beaty *et al.*, 2018a). Importantly, this intrinsic network connectivity profile was able to predict creative ability in three independent participant samples, with the density of default to executive coupling identified as the most predictive feature. The current study replicated this pattern of intrinsic network connectivity associated with creativity in our young cohort. Significant connections were observed between hubs of the DN and SN, as well as between the DN and the FPN, the DN and FPN and between the FPN and SN. These findings add to the growing body of evidence suggesting that default to executive coupling is an important neural marker of creative thought in young adults.

Building from these young adult findings, here we show that the intrinsic neural substrate of creative thought is altered in older adulthood, with the most prevalent differences observed as greater coupling between the default and the broader executive control network, including nodes of both the FPN and SN sub-components. Consistent with a recently proposed neurocognitive aging model of aging, DECHA (Turner and Spreng, 2015; Spreng *et al.*, 2018), we interpret these results to suggest that older adults may access and manipulate information contained in their store of crystalized knowledge to generate creative responses. Based on the DECHA model, this greater default–executive coupling in typical aging may support cooperation between activated prior knowledge representations, mediated by default brain regions, and executive control processes necessary to leverage these representations to construct novel associations. Consistent with this idea, reliance on mnemonic processes during creative cognition has been recently been reported for older adults (Madore *et al.*, 2016).

Our ability to detect creativity–RSFC associations during the resting state suggests that these age differences may be enduring and do not simply reflect changes in strategy or approach to the task. As the balance of cognitive resources shifts from controlled to crystalized capacities across the lifespan (Park *et al.*, 2001), we suggest that creativity becomes increasingly dependent on access to prior knowledge representations. With a lifetime of accumulated knowledge and experience, this engagement of prior knowledge in the service of goal-directed tasks reduces segregation between default and executive networks, with implications for multiple cognitive abilities. On tasks where prior knowledge is incongruent or distracting for task goals, greater default to executive coupling is associated with poorer task performance (e.g. Rieck *et al.*, 2017; Spreng *et al.*, 2018). Critically, however, when access to prior knowledge is goal congruent, default-executive coupling is associated with better performance, at least in young adults ( Spreng *et al.*, 2014; Beaty *et al.*, 2016). Here, we provide evidence that this pattern also holds for older adults as greater default–executive coupling was more robustly predictive of creative cognition for older adults. We recently reported a similar pattern of default–executive coupling in the domain of autobiographical memory, with more semanticized recall associated with a pattern of greater default to executive coupling in older but not younger adults (Spreng *et al.*, 2018).

Our findings also highlight the role of vmPFC, a core DN node, in creative cognition in older adults. We observed that greater intrinsic bilateral coupling of vmPFC, as well as stronger between-network connectivity to executive control nodes, specifically within the SN, was associated with creativity in our older participants. Our recent task findings also revealed greater coupling between vmPFC and the middle temporal gyrus, a region of the DN, during divergent thinking (Adnan *et al.*, 2019). While speculative, the involvement of this region may hint at an alternative pathway supporting creative thinking in later life. The vmPFC is a core hub of the DN and comprises the anterior, self-referential subsystem of the network (Andrews-Hanna *et al.*, 2010; Andrews-Hanna, 2012). Within-network connectivity of this region to medial temporal lobe subsystems as well as between-network connections with executive control regions (such as the temporal-parietal junction, insula, middle frontal gyri, supramarginal gyrus) has been implicated in accessing and engaging autobiographical knowledge to support goal-directed tasks (Andrews-Hanna *et al.*, 2014). Consistent with this idea, the vmPFC has recently been posited as a gateway node, controlling access to consolidated or more semanticized autobiographical memory (Bonnici and Maguire, 2018). Here, we suggest that access to one’s store of consolidated, or crystalized, experiential knowledge, reflected in the intrinsic connectivity patterns of the vmPFC, may be an important mechanism associated with creative cognition in later life.

Our findings suggest that intrinsic connectivity between the default and the executive control network (including both FPN and salience components) is associated with creative ability in later life. While default–executive coupling predicted divergent thinking ability in both young and older adults, the between-network connectivity pattern was more distributed and more robust for the older adult cohort. While these findings are broadly consistent with our recent task-based fMRI results (Adnan *et al.*, 2018), these intrinsic connectivity data suggest that between-network coupling is not solely a task-specific neural response but rather an entrained shift in the neural processes underlying creative thinking ability in later life. Moreover, we postulate that access to a comparatively preserved repertoire of stored personal knowledge and experiences in later life, reflected in greater within and between-network connectivity of the anterior DN, is associated with preserved creative thinking ability in older adulthood.

## Supplementary Material

nsz013_Supp.docx
