# Supplementary material for: Intrinsic default—executive coupling of the creative aging brain
Source: Soc Cogn Affect Neurosci. 2019 Feb 20;14(3):291–303. doi: 10.1093/scan/nsz013 (PMC6399613; doi:10.1093/scan/nsz013)
Supplement: nsz013_Supp.docx [file nsz013_supp.docx]

**Supplementary Materials**

**Methods**

*Participants*

22 old (mean age = 68.9 years, *SD* = 5.27; 11 females) and 22 young (mean age: 24.76, SD = 3.36;15 females) were included in a supplemental analysis matched for the personality trait openness to experience. As with the full sample reported in the main text, this subset of participants were recruited from the community and participated in a comprehensive cognitive test battery and magnetic resonance image (MRI) scanning as part of a larger ongoing study. Participants received monetary compensation for their time (equivalent to $50 for the MRI scan and $10 CAD per hour). To be eligible for the study, participants had to be: a) over age 60; b) right-handed; and c) a fluent English speaker. Exclusion criteria included any MRI contraindications and/or a history of neurological, neuropsychiatric, or cardiovascular disease. All participants provided informed consent consistent with procedures approved by the Institutional Review Board of York University. All participants were cognitively healthy based on subjective reports and had MMSE scores greater than 24.

Previous work has shown that both creative ability (Feist, 1998; McCrae, 1987; Silvia, Nusbaum, Berg, Martin, & O'Connor, 2009) and default network engagement (Beaty et al., 2018b) are predicted by the ‘Big-Five’ personality trait of ‘openness’. Given that our participants were a subsample of a larger ongoing study, we chose to match our participants on the personality trait of openness. Previous work has shown that intrinsic coupling between networks implicated in creativity are also related to openness to experience (Beaty et al., 2018) and that divergent thinking is associated with openness to experience (Feist, 1998; McCrae, 1987; Silvia, Nusbaum, Berg, Martin, & O'Connor, 2009). Thus, we chose to match our young and older adults on personality to ensure that between group differences were not due to differences in personality.

In the larger sample of participants, 22 young adults (mean age: 24.76, SD = 3.36; 15 females) and 44 older adults (mean age: 69.80, SD = 7.05; 24 females) had completed both the divergent thinking measures and a comprehensive personality inventory, the Big Five Aspect Scales (BFAS). A two-tailed t-test revealed that there was a significant difference in self-reported openness to experience between young (*M* = 3.52, *SD* = 0.26) and older adults (*M* = 3.81, *SD* = 0.24), *t* (64) = -2.19, *p* = 0.032, Cohen’s d = 1.16). Given prior work associated creativity and personality and the rationale set up above, we matched a subset of older adults to our young adult sample on the personality trait of openness to experience, based on Big-Five Openness (BFAS-O) scores. We obtained two groups with 22 young (mean age = 24.76 years, *SD* = 3.39) and 22 older adults (mean age = 68.9 years, *SD* = 5.27) who were then subsequently used in analyses and did not differ significantly BFAS-O scores, *t* (42) = -1.05, *p* = 0.29.

Analysis Approach

We took a within-group approach to examine creativity associated patterns of RSFC among our networks of interest in young and older adults. Here, we examined within group patterns of connectivity (young and older adults) independently, and offline measures of creativity were used as a second level regressor of interest in both analyses. Results were corrected for multiple comparisons using a false discovery rate threshold of 0.05, at the ROI level. For both groups, positive findings reflect patterns of ROI-to-ROI connectivity that positively correlate with creative ability, while negative findings indicate negative correlations with creative ability.

Second, to look at age related differences in creativity, we used a between group analysis. Here, we contrasted group level maps of ROI-to-ROI functional connectivity correlating with offline measures of creativity. This contrast was specified as *Older Adults > Young Adults.* Results were corrected for multiple comparisons using a false discovery rate threshold of 0.05, at the ROI level. For this analysis, positive findings reflect patterns of functional connectivity that correlate with creative ability in older adults; in contrast, negative findings indicate findings in young adults.

**Results**

*Within-Group Patterns of Functional Connectivity Associated with Creative Ability*

Young adults showed a distributed pattern of between-network functional connectivity that positively predicted divergent thinking performance outside of the scanner. Functional connectivity between nodes of the salience network (left anterior insula) and FPN (left inferior frontal gyrus) and between FPN (left inferior temporal gyrus) and default networks (right ventromedial PFC) positively correlated with creativity ratings on the divergent thinking task. Right dorsolateral PFC, a node of the FPN, was significantly connected to bilateral posterior cingulate cortex as well as left anterior cingulate cortex, nodes of the default network and positively predicted creative ability in the young adult cohort. Supplementary Table 1 and Supplementary Figure 2 provide details for these nodes and associated connectivity results. For older adults, both within- and a more spatially distributed between- network connectivity profile was positively associated with creative task performance. Within-network connectivity was observed between nodes of the salience network (left insula and left supplementary motor area). Between-network connectivity was observed among default (right middle temporal gyrus) and salience (right cingulate gyrus) network nodes as well as between salience network (supplementary motor area) and FPN (inferior temporal gyrus) nodes. Additional between-network connectivity that was positively associated with creativity was observed between nodes of the default network (right superior temporal gyrus) and the FPN (right superior frontal gyrus). Supplementary Table 2 and Supplementary Figure 3 provide details for these nodes and associated connectivity results.

*Age Differences in Patterns of Functional Connectivity Associated with Creative Ability*

When the brain and behavior correlation maps for both age groups were directly contrasted, a pattern of greater within-network connectivity was associated with better divergent thinking performance for the younger cohort. Specifically, greater connectivity between default network nodes, including between left ventromedial PFC and left superior frontal gyrus, left medial frontal gyrus, left superior temporal gyrus and right dorsomedial PFC was associated with better divergent thinking ability for the younger participants. Young adults also had greater functional connectivity between default and FPN nodes associated with better divergent thinking ability, specifically between left inferior precentral sulcus and bilateral posterior cingulate cortex and between left ventromedial PFC and right inferior frontal gyrus and posterior middle temporal gyrus.

In older adults, greater between-network functional connectivity was associated with better divergent thinking ability. Greater between-network functional connectivity, associated with better task performance, was also observed between salience and default networks (right ventromedial PFC and right intraparietal sulcus) as well as among nodes of the default, FPN and salience networks (left ventromedial PFC, right anterior PFC and right middle frontal gyrus). One significant within-network connection, between the left and right ventromedial PFC nodes of the default network, was correlated with better creative ability for older adults (Supplementary

Figure 4, Supplementary Table 3).

**Supplementary Figures**

**
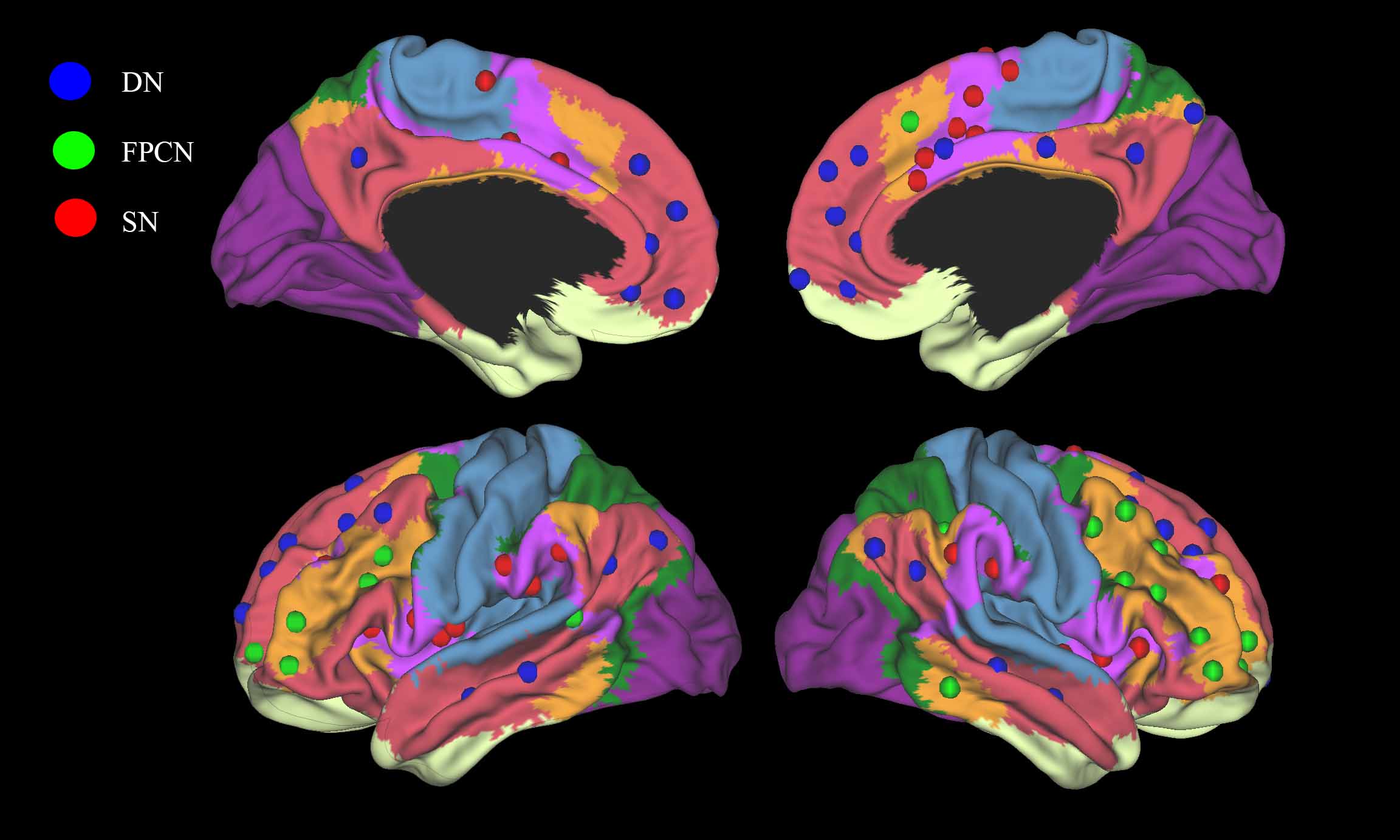
**

**Supplementary Figure 1.** Visualization of our networks of interest. Regions of Interest for the default, cingulo-opercular and frontoparietal control networks were taken from a resting state parcellation by Gordon and colleagues (2014) and are represented as spheres. For illustrative purposes, regions of interest were superimposed on an overlay (Yeo et al., 2011) to validate functional network assignment. The cingulo-opercular nodes from Gordon et al (2014) overlap with the salience network identified in Yeo at el (2011) and is referred to as the salience network in our analyses.

**
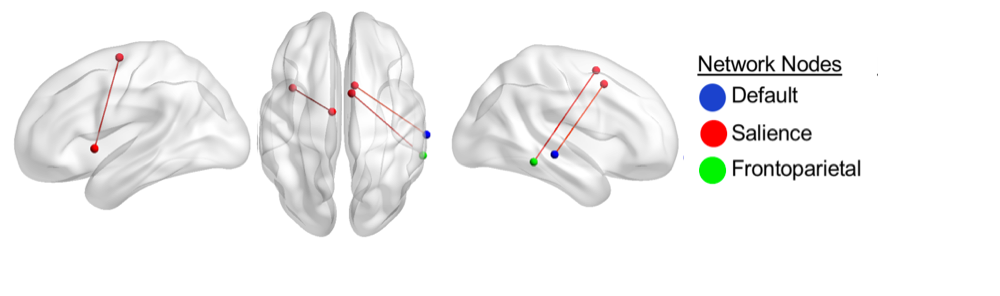
**

**Supplementary Figure 2.** Resting state functional connectivity correlating with divergent thinking ability in young adults. Color coded nodes include regions from the default network (DN), frontoparietal network (FPN) and salience network (SN). The color of the edges denotes the direction of correlation between functional connectivity and divergent thinking ability. Only positive correlations between ROI-to-ROI functional connectivity and divergent thinking ability survived a seed-level FDR correction at an alpha level of 0.05. Results correspond to findings in Supplementary Table 1.

**
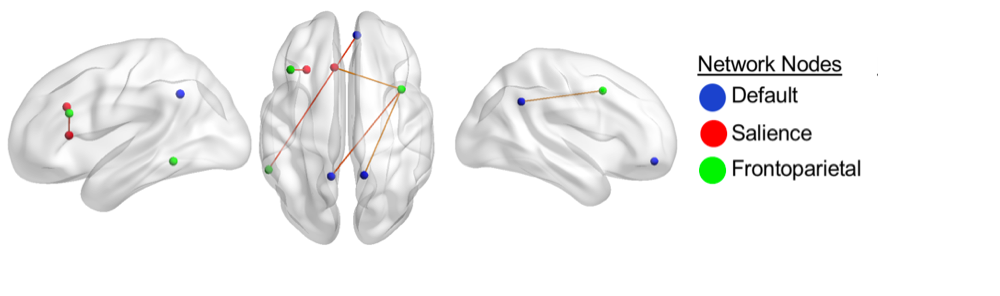
**

**Supplementary Figure 3.** Resting state functional connectivity correlated with divergent thinking ability in older adults (n = 22) matched with younger adults on the personality trait, openness to experience. Color coded nodes include regions from the default network (DN), frontoparietal network (FPN) and salience network (SN). The color of the edges denotes the direction of correlation between functional connectivity and divergent thinking ability. Only positive correlations between ROI-to-ROI functional connectivity and divergent thinking ability survived a seed-level FDR correction at an alpha level of 0.05. Results correspond to findings in Supplementary Table 2.

**
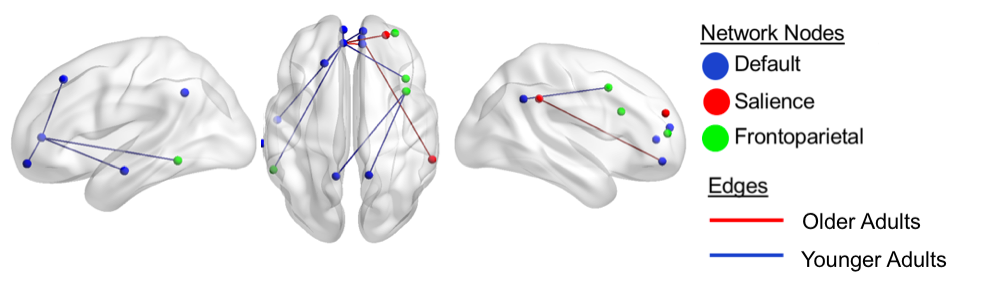
**

**Supplementary Figure 4.** Group by behavior interaction for intrinsic connectivity correlated with divergent thinking. The figure shows resting-state ROI-to-ROI functional connectivity that correlates with divergent thinking ability and is significantly different between young and personality-matched older adults. Color coded nodes include regions from the default network (DN), frontoparietal network (FPN) and salience network (SN). The color of the edges (connections between nodes) indicate the direction of the contrast. Red edges indicate greater connectivity between regions that are associated with divergent thinking in older adults, while blue edges indicate greater connectivity between regions that are associated with divergent thinking in young adults. Results correspond to findings in Supplementary Table 3.

**Supplementary Table 1.** ROI-to-ROI connectivity positively correlating with divergent thinking ability in young adults (corresponding to Supplementary Figure 2).

|  |  | ***Network*** | ***Hem*** | ***Node*** | ***MNI Coordinates*** | | | ***T*** | ***p*** |
| --- | --- | --- | --- | --- | --- | --- | --- | --- | --- |
|  |  |  |  |  | ***X*** | ***Y*** | ***Z*** |  |  |
| **Young Adults** | |  |  |  |  |  |  |  |  |
| ***Between Network Connectivity*** | | |  |  |  |  |  |  |  |
| *SN-FPN* |  |  |  |  |  |  |  |  |  |
| **Anterior Insula** | | SN | L | 84 | -28.8 | 23.7 | 8.4 |  |  |
|  | Inferior Frontal Gyrus | FPN | L | 109 | -43 | 19.4 | 33.5 | 4.24 | 0.04 |
| *FPN-DN* |  |  |  |  |  |  |  |  |  |
| **Inferior Temporal Gyrus** | | FPN | L | 9 | -55.9 | -47.7 | -9.3 |  |  |
|  | Ventromedial PFC | DN | R | 279 | 7.2 | 48.4 | -10.1 | 4.23 | 0.043 |
| *FPN-DN-SN* | |  |  |  |  |  |  |  |  |
| **Dorsolateral PFC** | | FPN | R | 328 | 38.9 | 9.6 | 42.7 |  |  |
|  | PCC | DN | L | 1 | -11.2 | -52.4 | 36.5 | 4.07 | 0.046 |
|  | Middle ACC | SN | L | 28 | -9 | 25.3 | 27.7 | 3.72 | 0.047 |
|  | PCC | DN | R | 162 | 12.3 | -51.6 | 34.5 | 0.74 | 0.047 |

Note: Hem = Hemisphere; SN - Salience Network; FPN - Fronto-parietal network; DN - Default Network; PFC - Prefrontal Cortex; PCC – Posterior Cingulate Cortex; ACC – Anterior Cingulate Cortex; R – Right; L – Left.

**Supplementary Table 2.** ROI-to-ROI connectivity positively correlating with divergent thinking ability in older adults (corresponding to Supplementary Figure 3).

|  |  | | ***Network*** | | ***Hem*** | ***Node*** | ***MNI Coordinates*** | | | ***T*** | ***p*** |
| --- | --- | --- | --- | --- | --- | --- | --- | --- | --- | --- | --- |
|  |  |  |  |  |  |  | ***X*** | ***Y*** | ***Z*** |  |  |
| **Older Adults** | | |  | |  |  |  |  |  |  |  |
| ***Between Network Connectivity*** | | | | |  |  |  |  |  |  |  |
| *DN-FPN* | |  | |  |  |  |  |  |  |  |  |
| **Superior Frontal Gyrus** | |  | | FPN | R | 277 | 28.4 | 57 | -5.1 |  |  |
|  | | Superior Temporal Gyrus | | DN | R | 290 | 57.5 | -7.4 | -16.4 | 3.78 | 0.0011 |
| *DN-SN* | |  | |  |  |  |  |  |  |  |  |
| **Cingulate Gyrus** | |  | | SN | R | 187 | 8.8 | 10.8 | 45.9 |  |  |
|  | | Middle Temporal Gyrus | | DN | R | 225 | 62.5 | -25.6 | -5.5 | 2.93 | 0.008 |
| *SN-FPN* | |  | |  |  |  |  |  |  |  |  |
| **Inferior Temporal Gyrus** | |  | | FPN | R | 170 | 59.7 | -41 | -10.9 |  |  |
|  | | Supplementary Motor Area | | SN | R | 181 | 6.7 | 5 | 55.9 | 3.09 | 0.006 |
| ***Within Network Connectivity*** | | | | |  |  |  |  |  |  |  |
| *SN-SN* |  | |  | |  |  |  |  |  |  |  |
| **Supplementary Motor Area** |  | | SN | | L | 34 | -8 | 8.7 | 62.9 |  |  |
|  | Insula | | SN | | L | 82 | -37.3 | 8.9 | -0.9 | 3.25 | 0.004 |

Note: Hem = Hemisphere; SN - Salience Network; FPN - Fronto-parietal network; DN - Default Network; PFC - Prefrontal Cortex; PCC – Posterior Cingulate Cortex; ACC – Anterior Cingulate Cortex; R – Right; L – Left.

**Supplementary Table 3.** ROI-to-ROI connectivity correlating with divergent thinking ability contrasted between young and personality-matched older adults (Older Adults > Young Adults). Hence, Positive *T* values reflect ROI-to-ROI functional connectivity predicting divergent thinking in older adults, while negative T values correspond to ROI-to-ROI functional connectivity predictive of divergent thinking in young adults (corresponding to Supplementary Figure 4).

|  |  | | **Network** | **Hem** | **Gordon et al (2014) Node** | ***MNI Coordinates*** | | | ***T*** | ***p*** |
| --- | --- | --- | --- | --- | --- | --- | --- | --- | --- | --- |
|  |  |  |  |  |  | ***X*** | ***Y*** | ***Z*** |  |  |
| **Young Adults** | | |  |  |  |  |  |  |  |  |
| ***Between Network Connectivity*** | | | |  |  |  |  |  |  |  |
| *FPN – DN* | | |  |  |  |  |  |  |  |  |
| **Inferior Precentral Sulcus** | | | FPN | L | 328 | 38.9 | 9.6 | 42.7 |  |  |
|  | PCC | | DN | R | 162 | 12.3 | -51.6 | 34.5 | -3.99 | 0.0276 |
|  | PCC | | DN | L | 1 | -11.2 | -52.4 | 36.5 | -3.89 | 0.0188 |
| **Ventromedial PFC** | | | DN | L | 152 | -6 | 44.9 | 6.3 |  |  |
|  | Inferior Frontal Gyrus | | FPN | R | 276 | 38.6 | 18.8 | 25.5 | -3.93 | 0.0328 |
|  | Posterior Middle Temporal Gyrus | | FPN | L | 9 | -55.9 | -47.7 | -9.3 | -3.15 | 0.042 |
| ***Within Network Connectivity*** | | | |  |  |  |  |  |  |  |
| *DN-DN* | | |  |  |  |  |  |  |  |  |
| **Ventromedial PFC** | | | DN | L | 152 | -6 | 44.9 | 6.3 |  |  |
|  | Medial Frontal Gyrus | | DN | L | 116 | -5.9 | 54.8 | -11.3 | -3.64 | 0.0337 |
|  | Superior Frontal Gyrus | | DN | L | 44 | -19.5 | 30.1 | 45.4 | -3.55 | 0.0337 |
|  | Superior Temporal Gyrus | | DN | L | 127 | -53.1 | -11.4 | -16 | -3.39 | 0.0351 |
|  | Dorsomedial PFC | | DN | R | 322 | 8.2 | 53.8 | 14 | -3.04 | 0.047 |
| **Older Adults** |  | |  |  |  |  |  |  |  |  |
| ***Between Network Connectivity*** | | | |  |  |  |  |  |  |  |
| *SN-DN* | |  |  |  |  |  |  |  |  |  |
| **Intraparietal Sulcus** | | | SN | R | 219 | 57.5 | -40.3 | 34.7 |  |  |
|  | Ventromedial PFC | | DN | R | 279 | 7.2 | 48.4 | -10.1 | 4.35 | 0.0092 |
| *DN-FPN-SN* | | |  |  |  |  |  |  |  |  |
| **Ventromedial PFC** | | | DN | L | 152 | -6 | 44.9 | 6.3 |  |  |
|  | Anterior PFC | | FPN | R | 320 | 30.9 | 52.2 | 9.9 | 3.36 | 0.0351 |
|  | Middle Frontal Gyrus | | SN | R | 317 | 24.4 | 50.8 | 24.3 | 3.22 | 0.042 |
| ***Within Network Connectivity*** | | | |  |  |  |  |  |  |  |
| *DN-DN* | | |  |  |  |  |  |  |  |  |
| **Ventromedial PFC** | | | DN | L | 152 | -6 | 44.9 | 6.3 |  |  |
|  | Ventromedial PFC | | DN | R | 184 | 7.7 | 44.1 | 5.5 | 3.13 | 0.042 |
|  |  | |  |  |  |  |  |  |  |  |

Note: Hem = Hemisphere; SN - Salience Network; FPN - Fronto-parietal network; DN - Default Network; PFC - Prefrontal Cortex; PCC – Posterior Cingulate Cortex; ACC – Anterior Cingulate Cortex; R – Right; L – Left.
